# Polyurethane‐polypyrrole hybrid structural color films for dual‐signal mechanics sensing

**DOI:** 10.1002/SMMD.20220008

**Published:** 2022-12-23

**Authors:** Changmin Shao, Yunru Yu, Qihui Fan, Xiaochen Wang, Fangfu Ye

**Affiliations:** ^1^ Oujiang Laboratory (Zhejiang Lab for Regenerative Medicine, Vision and Brain Health) Wenzhou Institute University of Chinese Academy of Sciences Wenzhou Zhejiang China; ^2^ Beijing National Laboratory for Condensed Matter Physics Institute of Physics Chinese Academy of Sciences Beijing China; ^3^ School of Physical Sciences University of Chinese Academy of Sciences Beijing China

**Keywords:** dual‐signal, mechanics monitoring, photonic crystal, polypyrrole, structural color

## Abstract

The monitoring of mechanical indexes involved in body movement has attracted immense interest in the diagnosis of neurodegenerative diseases. Here, we present a hybrid flexible conductive structural color (SC) film with the capability of dual‐signal mechanics screening. The film is constructed by oxidatively polymerizing pyrrole on the surface of an inverse opal polyurethane (IPU) membrane, which can be utilized to measure the mechanical indexes through resistance change. Owing to the inverse opal structure, the film shows visual structural color change when stretched and released according to the body movement. Additionally, the highly uniform ordered porous structure endows the conductive film with a lower coefficient of variance on relative resistance change. Benefiting from these features, we have demonstrated that such a flexible conductive SC film could monitor Parkinson's disease (PD) by detecting mechanical indexes simultaneously via dual signals. These features indicate the great value of the stretchable conductive SC films in mechanics sensing applications.

## Graphical abstract

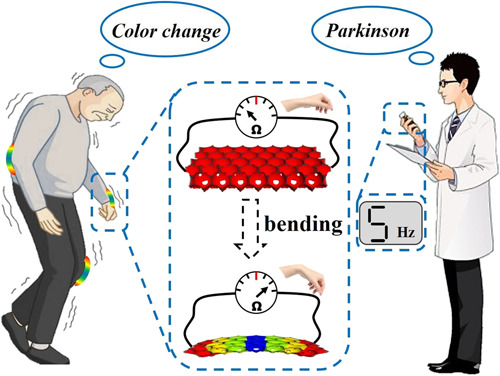

1

**Key points**

- A hybrid flexible conductive structural color (SC) film is fabricated for dual‐signal mechanics screening.
- The polyurethane (IPU) on the film can be utilized to measure the mechanical indexes through resistance change.
- The structural color change of the film during stretching can be used to monitoring the body movement.
- The as‐prepared hybrid film can monitor Parkinson's disease (PD) by detecting mechanical indexes simultaneously via dual signals.

## 2 INTRODUCTION

Health has always been a permanently and closely concerned topic, which is strongly related to human development and progress.^1^, ^2^, ^3^ In addition to the conventional physiological indicators, such as blood glucose, blood pressure, and biomarkers, etc., a wide variety of mechanics indexes related to the muscle, skeleton, and ligament are still in great demand to be monitored and measured, involving in the rehabilitation medicine, preventive medicine, sanipratics, clinical medicine, and sports medicine.^4^, ^5^, ^6^, ^7^, ^8^ Currently, plenty of large‐scale instruments have been developed for capturing the mechanical indexes and analyzing their physiological stability, variation tendency, and abnormal fluctuations, which are utilized to assess the physical health condition and predict some specific diseases. However, these instruments are usually costly, bulky, and need professionally trained technicians and cannot be adopted as household devices to monitor daily life activities and health status, especially for the aged.^9^, ^10^, ^11^ In addition, the detected signals of the existing instruments for mechanics index monitoring, such as electrical signals and color signals, are simplex and not sensitive enough, which restricts their practical applications in life and the health field to a large extent.

**Scheme 1:**
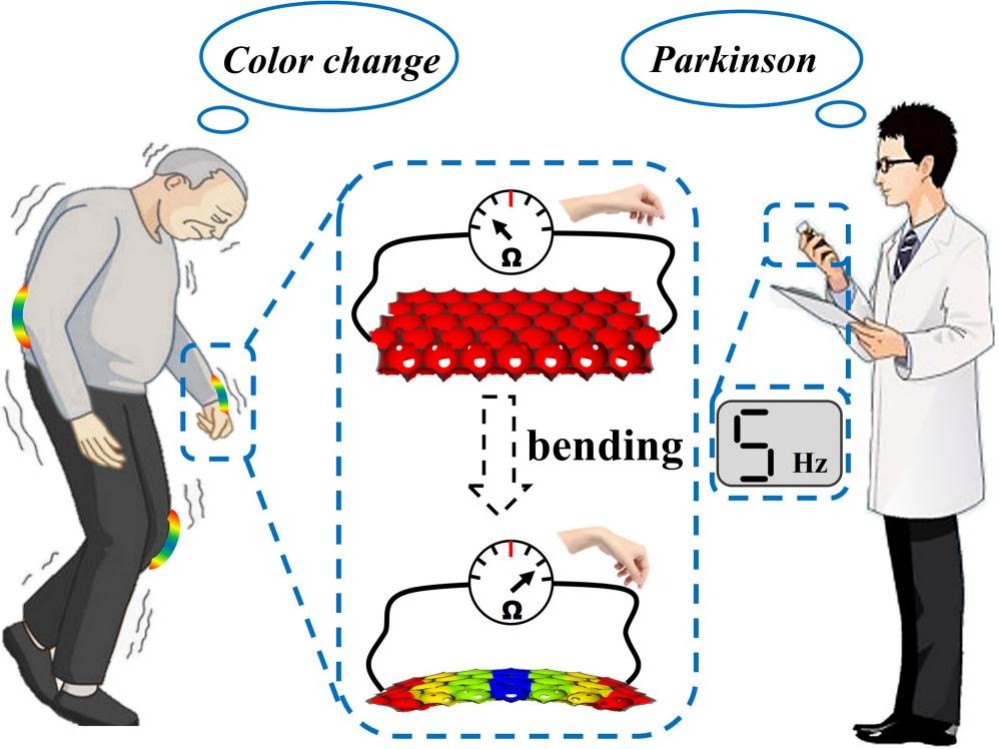
Schematic diagram of the structural color films for dual‐signal mechanics sensing.

In this work, we propose a novel hybrid flexible film with inverse opal structure and conductive functionality to screen dual‐signal mechanics, as shown in Scheme 1. Structural color (SC) material is a kind of metamaterial composed of different components with their respective specific dielectric constant and periodically spatial arrangement.^12^, ^13^, ^14^, ^15^, ^16^, ^17^ Owing to the photonic bandgaps resulting from the periodic modulation of the refractive index of the host materials and the surrounding environment, the SC materials present vivid structural colors, which can thus be expediently and efficiently tailored by varying the periodic structure and refractive index of materials.^18^, ^19^, ^20^, ^21^, ^22^, ^23^, ^24^ Therefore, the SC productions have been widely adopted as sensors for bio‐detection, environmental monitoring, bio‐safety, as well as flexible electronics through their visual color change.^25^, ^26^, ^27^, ^28^, ^29^, ^30^ However, the single signal transfer mode of these sensors heavily depends on the performance of the material and could easily be disturbed by the external environment and multifarious application scenarios.^31^, ^32^, ^33^, ^34^, ^35^ In addition, there remain challenges to effectively combine SC and conductive materials to integrate the optical signal with the electrical signal for dual‐signal detection, especially for flexible electronics‐based mechanical sensing.^36^, ^37^, ^38^, ^39^, ^40^ Thus, there is great expectation to create brand new composite films coalescing the visualization feature of SC and electrical conductivity function for mechanical sensing with enhanced accuracy.

Herein, we develop a stretchable conductive SC film by polymerizing pyrrole on the surface of an inverse opal polyurethane (IPU) film for dual‐signal mechanics sensing. Polypyrrole (PPy) is a commonly used conductive polymer, which is synthesized by polymerizing pyrrole monomer with the oxidizing agent.^41^, ^42^, ^43^, ^44^, ^45^ The in situ oxidized and polymerized PPy on the inverse opal scaffold endowed the SC film with a conductive property, whose resistance change during the body motion could be utilized to measure the related mechanics indexes. In addition, the change of structural colors induced by the stretching and releasing process of this flexible IPU SC film could not only visually monitor body motion but also quantificationally detect the mechanical indexes. Moreover, benefiting from the highly ordered and uniform inverse opal structure, the film presented a much lower coefficient of variance (CV) on relative resistance change compared with that composed of random pores. It was demonstrated that such a hybrid flexible conductive SC film could simultaneously detect two signals, electrical signal plus optical signal, and accurately sense body movement, and it has great potential for monitoring neurodegenerative diseases, such as Parkinson's disease (PD). PD is a severe neurodegenerative disease characterized by progressive symptoms of static tremor, rigidity, bradykinesia, and gait freezing.^46^ Continuous deterioration can result in executive dysfunctions, memory disturbances, and reduced ability to smell, which will seriously affect the life quality.^47^, ^48^, ^49^ There are mainly two diagnostic methods for the assessment of PD, including biomarker detection and wearable sensing.^50^, ^51^, ^52^ However, these methods cannot satisfy the demand of accuracy and convenience simultaneously. Here, we demonstrated a dual‐signal detection of PD using the SC film through a modified model. These features indicated the practical applicability of the flexible conductive SC films in the field of mechanics sensing.

## 3 RESULTS AND DISCUSSION

**FIGURE 1:**
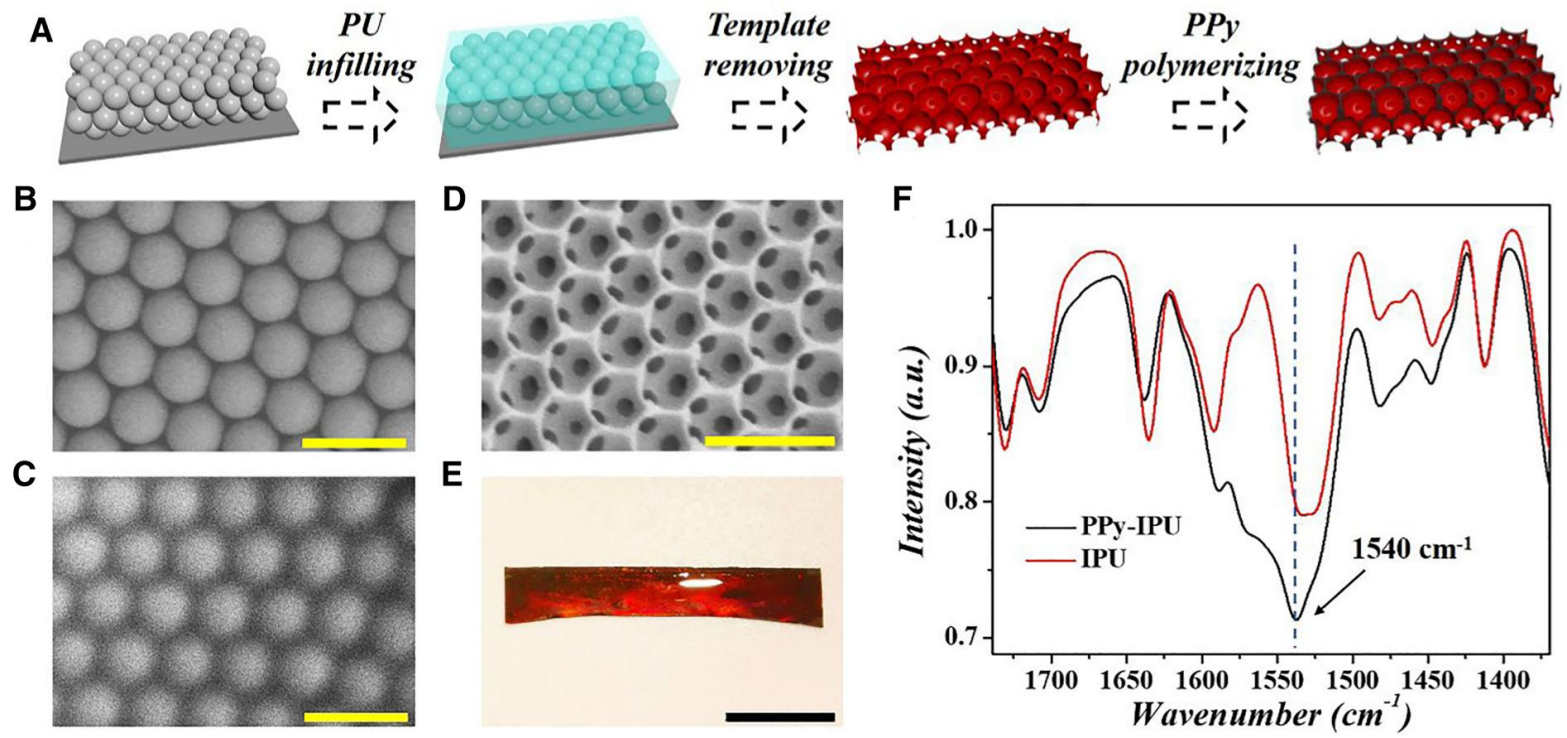
Overview of the polyurethane–polypyrrole hybrid structural color film. (A) Scheme illustrated the fabrication of the conductive SC film. (B–D) SEM images of the (B) silica colloidal crystal template, (C) the PU infiltrated colloidal crystal, (D) the IPU. (E) Optical image of the conductive SC film. (F) FTIR spectra of the PPy, indicated by the green dotted box. Scale bars are 500 nm in (B), (C), (D) and 1 cm in (E).

In a typical experiment, an IPU film was prepared by duplicating silica colloidal crystal templates via the ordered self‐assembly of silica colloidal nanoparticles using the doctor blade coating method (Figure 1A). In this method, the silica colloidal nanoparticles were first arranged in an ordered face‐centered cubic close‐packed array after heating‐induced solvent evaporation. Then the colloidal crystal templates were further calcined for 4 h at 600°C to strengthen the close‐packed structure (Figure 1B). The hybrid PU‐silica film was obtained by adding the PU solution with a concentration of 20% (w/v) onto the templates, which could infiltrate into the voids of the templates with the assistance of capillary force, and evaporating the solvent to solidify it (Figure 1C). Then, the hybrid film was immersed into HF solution (4%, v/v) to remove the silica templates. Finally, an IPU film was acquired by thoroughly rinsing it with deionized water (Figure 1D). A PU film with a random pore structure was fabricated using similar methods by polydisperse silica colloidal nanoparticles with different diameters (Figure S1).

To impart the IPU film with the capability of electrical signal collecting, PPy was grafted onto the IPU film by chemical oxy‐polymerization (Figure S2). In this procedure, the hydroxyl group was first introduced onto the film surface via oxygen plasma, then a conventional chemical vapor deposition method was adopted to graft *N*‐(3‐Trimethoxysilylpropyl) pyrrole monomer onto the film. Sequentially, the film was treated with the pyrrole monomer and catalyst to trigger the oxy‐polymerization on the surface of the film by immersing it in a reaction solution. Owing to the interconnected porous network, the solution could infiltrate into the nanopores of the scaffold so that the PPy could be deposited on the whole surface of the inverse opal structure, not only the outer surface of the film. The polymerization of PPy on the IPU film could be confirmed by the appearance of the black background (Figure 1E). In addition, the FTIR (Fourier transform infrared) spectra also demonstrated the synthesis of the PPy through a characteristic peak of the antisymmetric ring stretching modes of PPy at 1540 cm^−1^ (Figure 1F).

The ordered arrangement of nanopores imparted the resultant IPU film with specific optical properties. To be specific, the periodic alignment of air nanopores in the PU scaffold produced a photonic bandgap (PBG) with a definite wavelength, in which light with the same wavelength would be forbidden to pass through and reflected to present a vivid structural color. Under the circumstance of the incident light in a normal direction, the reflection peak value is usually calculated using Bragg's Law:

(1) $$\lambda =1.633dn_{\text{avg}}$$

in which *λ* indicates the wavelength value of the reflection peak, *d* refers to the diameter of the nanoparticles or nanopores, and *n*_avg_ presents the average refractive index of the matrix. In this case, according to Equation (1), the wavelength *λ* of the reflection peak along with the structural color could be easily regulated by alerting the colloidal nanoparticle diameter. Consequently, the IPU films demonstrating varied structural colors and corresponding reflection wavelengths could be obtained by replicating colloidal crystal templates assembled using silica nanoparticles of different diameters (Figure S3a). It was worth noting that after the oxy‐polymerization of the conductive PPy on the surface, the reflection peak of the obtained film showed a slight redshift, which might be attributed to the increase of the refractive index (Figure S3b). In addition, the polymerized black PPy on the conductive SC film provided a high contrast, making the film show a more vivid structural color than the nude IPU film.

**FIGURE 2:**
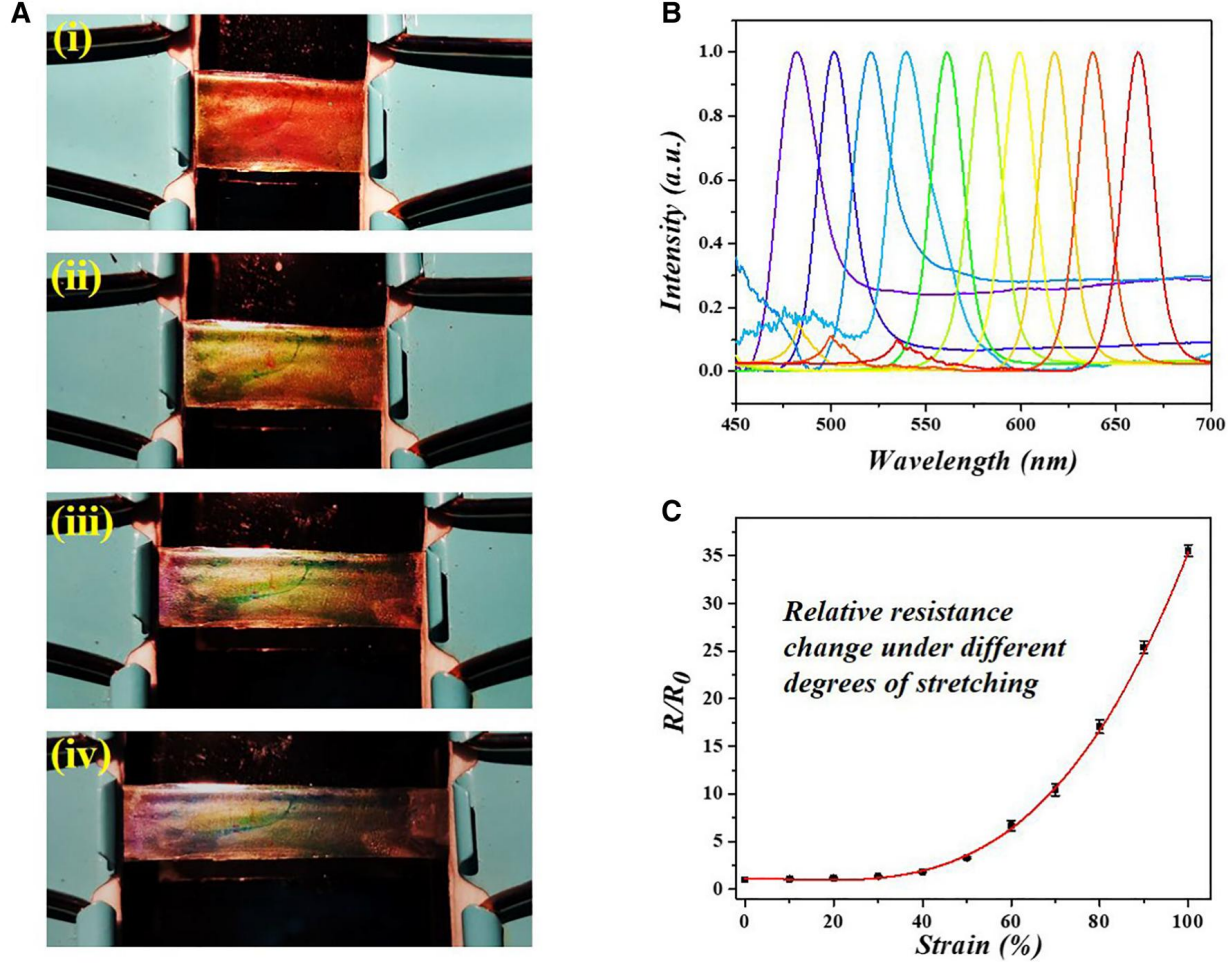
Optical and electrical response to the stretching. (A) Optical pictures of the structural color change of the conductive SC film. (B) The reflection wavelength blue‐shifting during the stretch of the conductive SC film. (C) The relative resistance change during the stretch of the conductive SC film.

PU is a widely used organic polymer with such dramatic stretchability that it has great potential for the creation of wearable devices. As an attribute of the inverse opal structure, the conductive SC film could exhibit an obvious structural color change during the stretching process. Specifically, along with the film stretching to 200% of its original length, the red structural color of the IPU film gradually changed to purple‐blue, as shown in Figure 2A and Movie S1. Accordingly, the reflection peak wavelength also exhibited an obvious blueshift from 661 to 482 nm due to the decreased diameter of nanopores *d* [Equation (1)], coming from the deformed nanopores in the IPU film (Figure 2B). Thus, by detecting the reflection peak wavelength of the IPU film during stretching, we could get the elongation degree of the film and quantitatively analyze the mechanics indexes applied to the film. The aforementioned optical characteristics proved that the obtained IPU film possessed potential value as an optical sensing device benefiting from its visible and measurable color change.

Except for the optical signal, the conductive SC film also presented the capability of electrical signal detection through the resistance changes with the graft of conductive PPy. For quantitative determination of the film elongation, the relative resistance change (*R*/*R*_*0*_, where *R* represents the real‐time resistance and *R*_*0*_ the original resistance) of the conductive SC film was measured when the film was stretched from its initial state to 200% length (Figure 2C). A remarkable change in *R*/*R*_*0*_ of the conductive SC film was detected with the increase of strain, which indicated effective electrical responsiveness against the large scope deformation. In addition, it was worth noting that profiting from the excellent elasticity of the PU polymer, the resistance change of the hybrid film during its stretching and releasing was also reversible. As indicated in Figure S4, the *R*/*R*_*0*_ was certified stable and repeatable during film stretching and loosening at 100% tensile strain with 200 repetitive cycles, showing robust durability of electrical signal detection during repeated deformation of the conductive SC film. The visual structural color and rapid electric response to the deformation of the film imparted the IPU film with excellent dual‐signal monitoring potential for mechanics sensing.

To further confirm the electrical property, the conductive performance of films experiencing different polymerization times of PPy on the inverse opal scaffold was first investigated. Figure S5a showed that the resistance of the IPU film declined with the increasing polymerization time, indicating that more PPy polymerized on the inverse opal scaffold resulted in better conductivity. Furthermore, the IPU films with different pore sizes replicated from different sized silica colloidal nanoparticles were tested to investigate the effect of nanostructure on the conductivity. The relative resistance change *R*/*R*_*0*_ versus tension of the different porous IPU films was shown in Figure S5b. The resistance of the prepared film changed along with the elongation of the IPU film, and the slopes of the curves in Figure S5b indicated that the sensitivity of the sensors fabricated with larger pores was higher than that with the smaller ones. This was because the sensors with larger pores underwent larger deformation under the same tensile force compared to those with smaller pores and presented a greater resistance change.

**FIGURE 3:**
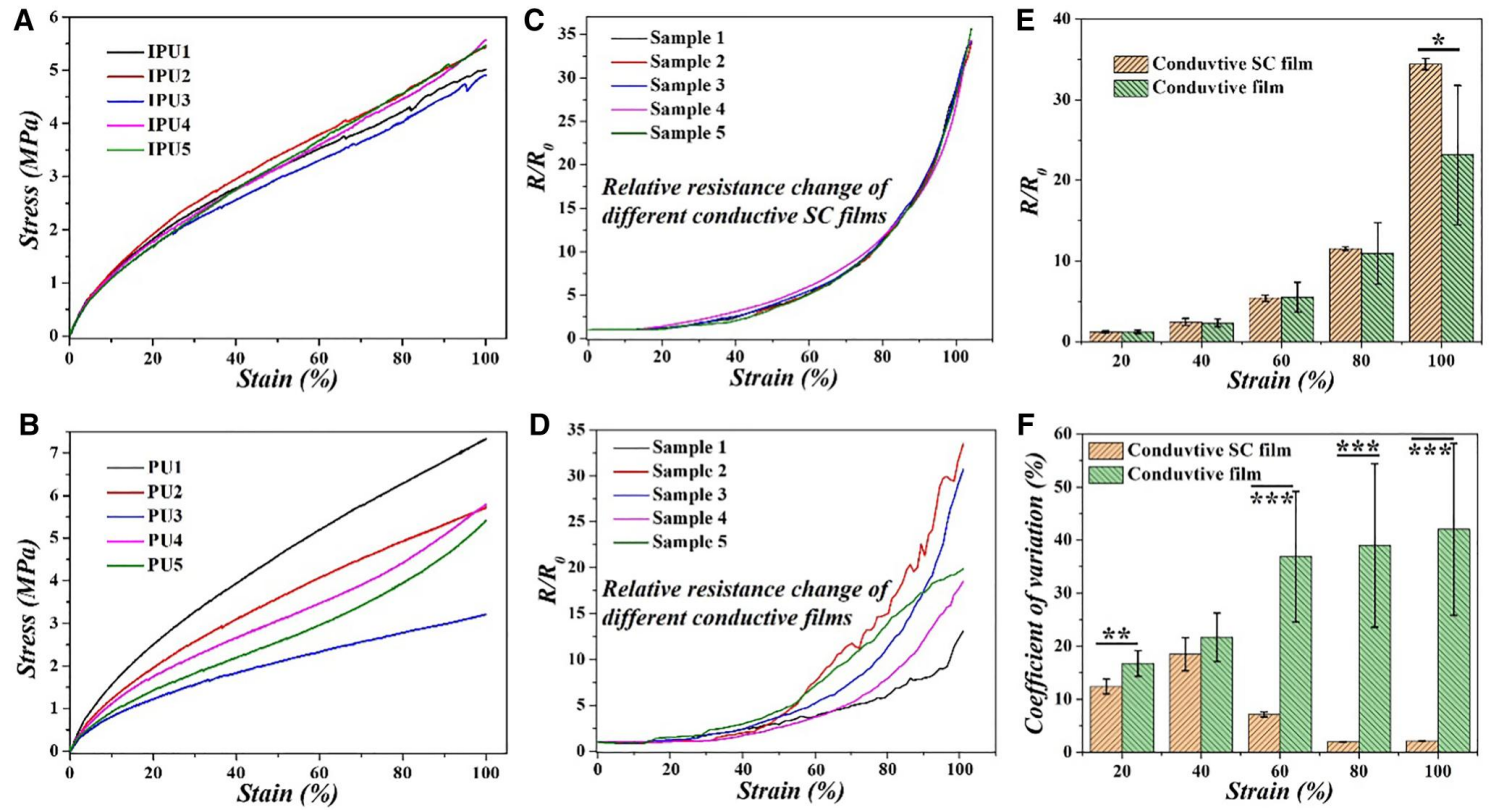
Uniformity in tensile strain and conductive property of the conductive SC film. (A, B) Stress–strain curves of five different films with (A) uniform pores and (B) random pores. (C, D) The relative resistance change vs. tensile curves of five different films with (C) uniform pores and (D) random pores. (E) Average relative resistance change at different stages of elongation of the conductive SC film and conductive film. (F) Coefficient of variation of relative resistance change at different stages of elongation of the conductive SC film and conductive film. Five samples were analyzed for *R/R*_*0*_ and CV. *p < 0.05, **p < 0.01, ***p < 0.001.

The uniform pore size and ordered arrangement of the inverse opal structure of the IPU film not only imparted the film with alterable structural colors but also endowed it with superior uniformity in both tensile strain and resistance change. As shown in Figure 3A, the stress–strain of five different IPU films fabricated from the silica templates with the same size was highly consistent, while there presented significant differences among the sensors that possessed random pores (Figure 3B). Accordingly, the resistance change of the IPU films also showed great repeatability. It could be seen that compared to the films with random pore structures, the five sensors replicated from same‐sized colloidal crystal templates showed very small variation in relative resistance change during stretching (Figure 3C,D). This could also be demonstrated by the average *R*/*R*_*0*_ and CV at different stages of elongation, respectively, in Figure 3E,F. For the IPU films, the CV was as low as 2%, while the CV of the conductive films with random pores exhibited a great difference in the stretching period. This excellent repeatability made the IPU film a promising candidate for durable mechanics sensing.

**FIGURE 4:**
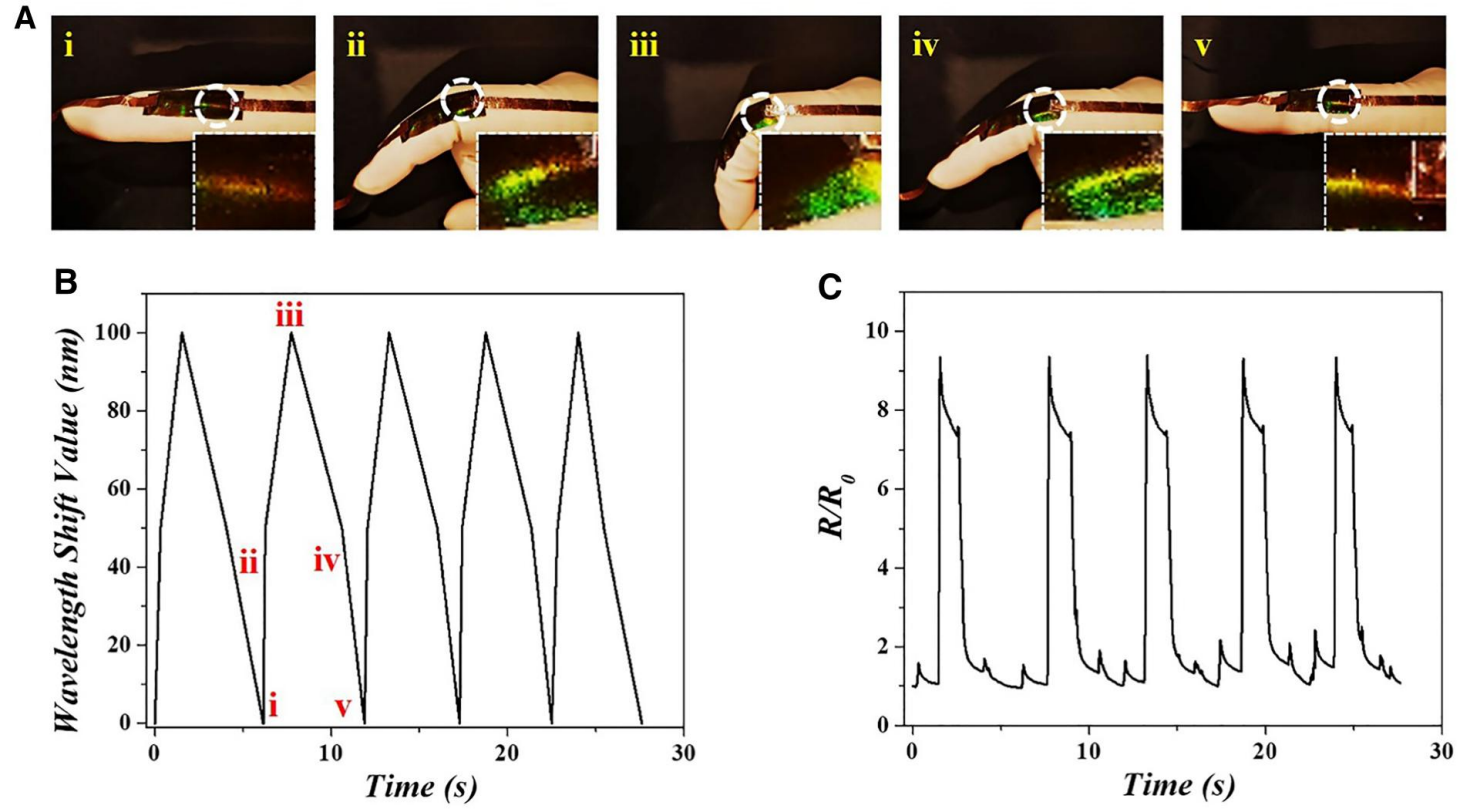
Dual‐signal monitoring of finger bending. (A) Optical images of the structural color change of the conductive SC film in response to the finger bending, the red dotted circle indicated the variation region at the joint. (B) The wavelength shift values of the conductive SC film in response to different bending states of a finger. (C) The relative resistance change of the conductive SC film during finger bending.

In light of the highly repeatable performance in both conductivity and stretchability, as well as the capability of visible structural color change, the IPU film was first applied to monitor the joint motions of the finger as a paradigm to realize mechanics sensing (Figure 4A). It is obvious that the structural color of the IPU film at the joint gradually changed to green from red and could return to the original color again when relaxed. In the repeated bending and relaxation of the finger, the IPU film presented the relevant color change as well as detectable wavelength shift value at the corresponding bending state (Figure 4B and Figure S6a). Simultaneously, the real‐time resistance of the conductive SC film was also detected (Figure 4C). It could be found that the relative resistance change of the film showed concurrent variations during the finger bending. More importantly, the *R/R*_*0*_ exhibited high uniformity in each repeat cycle (Figure 4C and Figure S6b). These results demonstrated that the optical and electrical signals of the fabricated conductive SC film presented a rapid and repeatable response to the dynamic bending motion of the finger, which showed sensitivity and durability in mechanics sensing.

**FIGURE 5:**
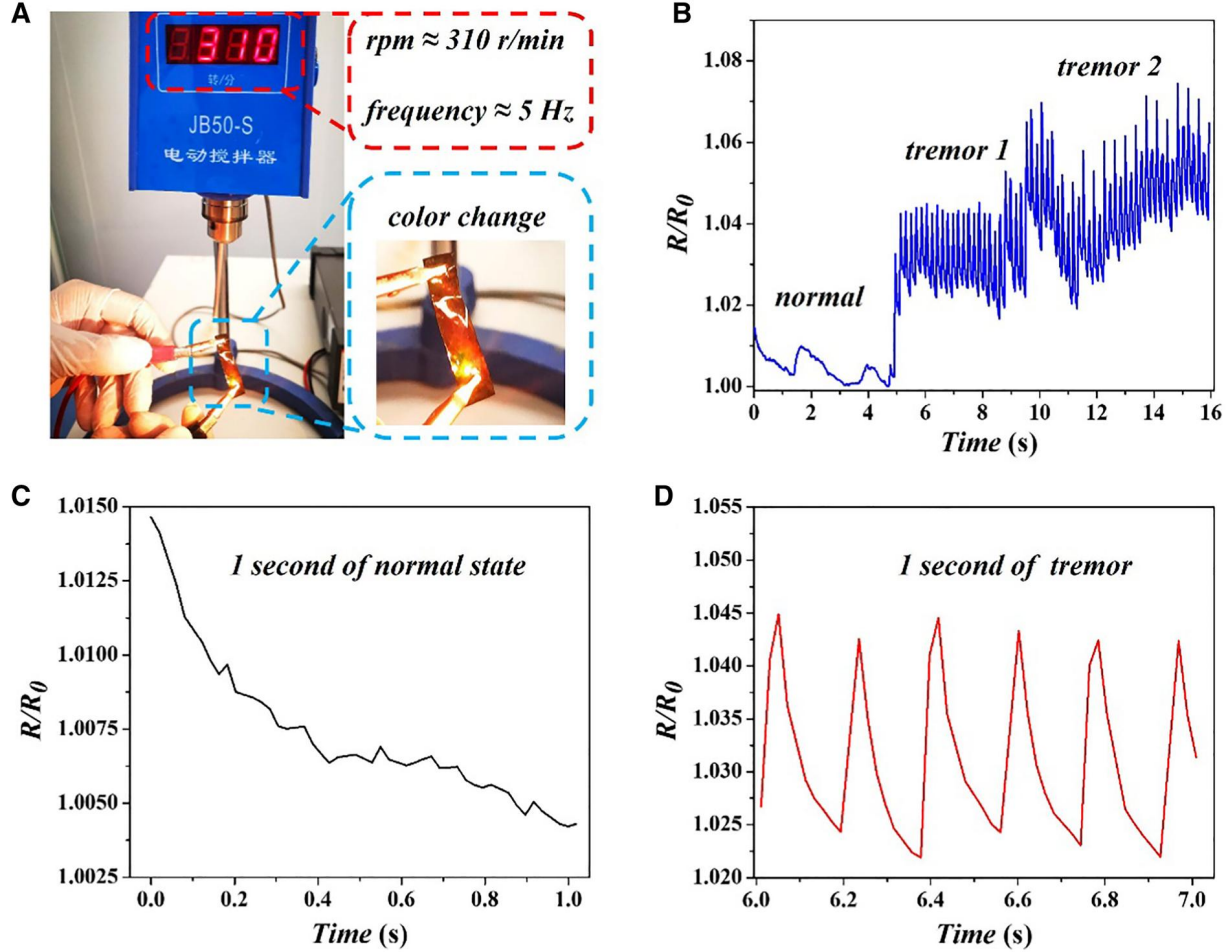
Early diagnosis of Parkinson's disease. (A) Picture of dual‐signal monitoring for Parkinson's disease tremor, simulated by a converted motor stirrer with a frequency of 5–6 Hz. (B) The relative resistance change of normal state and tremors with different intensities induced by applied force. (C) Detail of relative resistance change at normal state. (D) The relative resistance change of tremor state within 1 s, indicating a frequency of 5–6 Hz of the tremor.

Benefitting from these outstanding properties, the conductive SC film could show its potential in monitoring PD by detecting mechanical indexes. Considering that tremor with a frequency of 4–6 Hz is a typical symptom of PD at an early stage and can be adopted as a diagnosis proof, we applied the conductive SC films for quantitatively dual‐signal monitoring PD through a periodic ∼5 Hz disturbance (Figure 5A). As indicated in Figure 5B–D, the resistance of the conductive SC film maintained an irregular and slight fluctuation in a normal state, while experiencing a dramatical resistance change after applying the simulative tremor. Furthermore, the pulse amplitude varied with the strength of the applied force. It was obvious that the detected resistance of the SC film responded quickly to the deformation, and the change showed a frequency of 5–6 Hz, which possessed the capability for monitoring the tremor symptom of PD via the electrical signal. Moreover, the structural color of the conductive SC film transferred to yellow from the original red in response to the partial deformation of the film, which possessed the potential to optically monitor the body tremor in a static state. It is believed that with the help of a well‐established color library based on the measured reflect wavelength, the local color change of the SC film deformation could be transferred to the optical signal. In addition, to realize translational application, a set of systemic devices should be developed to integrate the SC film, electrical signal collector and optical signal detector, as well as a program for signal processing. We hope that the conductive SC films can play a role in the early diagnosis and dual‐signal assessment of PD.

## 4 CONCLUSION

In summary, we developed a composite stretchable SC film by integrating conducting polymer PPy with the IPU film for dual‐signal mechanics sensing. The obtained conductive SC film replicated from silica colloidal crystal template not only showed bright structural color but also had excellent electrical conductivity after in situ oxidative polymerization of PPy. The resistance change from PPy can be utilized to measure the mechanical indexes along with the stretching and releasing of the film during the movement. In addition, the excellent stretchability and inverse opal structure endow the IPU film with visually alterable structural color change during the elongation and recovery of the film, which can be utilized as an optical signal for mechanics sensing involved in body motions. Attractively, the IPU film is imparted with a lower CV on relative resistance change due to the highly ordered and uniform pores of the inverse opal structure. We have demonstrated that such a composite flexible SC film could monitor PD by detecting mechanical indexes simultaneously through both electrical and optical signals. These features make the composite flexible SC films suitable for flexible electronics focusing on mechanics sensing.

## 5 EXPERIMENTAL SECTION

Experimental details are provided in the Supporting Information.

## AUTHOR CONTRIBUTIONS

Fangfu Ye conceived the idea and designed the experiment; Changmin Shao conducted experiments and data analysis; Changmin Shao, Yunru Yu, Qihui Fan, Xiaochen Wang and Fangfu Ye wrote the manuscript.

## CONFLICTS OF INTEREST

The authors declare no conflict of interest. Fangfu Ye is a member of the *Smart Medicine* editorial board.

## ETHICS STATEMENT

The sensing experiments were carried out with the consent of healthy volunteers.

## Supporting information

Supplementary Material S1

Supplementary Material S2

## Data Availability

The data that support the findings of this study are available from the corresponding author upon reasonable request.
