# Supplementary material for: Polyurethane‐polypyrrole hybrid structural color films for dual‐signal mechanics sensing
Source: Smart Med. 2022 Dec 23;1(1):e20220008. doi: 10.1002/SMMD.20220008 (PMC11235726; doi:10.1002/SMMD.20220008)
Supplement: Supplementary file 1 — Supplementary Material S1 [file SMMD-1-e20220008-s002.docx]

Supplementary Information

Polyurethane-polypyrrole hybrid structural color films for dual-signal mechanics sensing

Changmin Shao ^1^, Yunru Yu ^1^, Qihui Fan ^1,2,^*, Xiaochen Wang ^1,2,3,^*, Fangfu Ye ^1,2,3,^*

^1^ Oujiang Laboratory (Zhejiang Lab for Regenerative Medicine, Vision and Brain Health), Wenzhou Institute, University of Chinese Academy of Sciences, Wenzhou, Zhejiang 325000, China.

^2^ Beijing National Laboratory for Condensed Matter Physics, Institute of Physics, Chinese Academy of Sciences, Beijing 100190, China.

^3^ School of Physical Sciences, University of Chinese Academy of Sciences, Beijing 100049, China.

Experimental Section

*Materials*

Silica colloidal nanoparticles with diameters of 215, 225, 237, 260, and 290 nm were prepared according to the Stöber method with appropriate modification. PU was purchased from Zhe Jiang Huafeng Thermoplastic Polyurethane Co., Ltd. Pyrrole and Iron (III) p-toluene sulfonate hexahydrate were purchased from Shanghai Yuanye Bio-Technology Co., Ltd. N-(3-trimethoxysilylpropyl) pyrrole was obtained from Alfa Aesar.

*Preparation of IPU films*

The colloidal crystal templates composed of silica were prepared through a doctor blade coating method on glass slides. The coating procedure was performed multiply to guarantee enough thickness. The acquired colloidal crystal templates were further calcined for 4 h at 600 ℃ to strengthen the close-packed structure. Then DMF solution containing 20% (w/v) PU was added onto the templates and allowed the solution to infiltrate into the voids of the obtained colloidal crystal. After solidification by heating at 80 °C, the IPU films were finally obtained by being etched in HF (4%, v/v) solution and rinsed with deionized water.

*Synthesis of PPy on the IPU films*

The prepared IPU films were first treated by oxygen plasma. Then the IPU films were set in a sealed container with a glass petri dish of 20 μL of N-(3-trimethoxysilylpropyl) pyrrole inside. The films were heated at 60 °C for 60 min to launch chemical vapor deposition of pyrrole (Py) graft on the IPU films. The Py monomer solution at the concentration of 3% (v/v) was mixed with an equal volume of Iron(III) p-toluene sulfonate hexahydratein catalyst solution at the concentration of 2.08% (w/v). Then the Py grafted IPU films were immersed in the mixture solution to undergo an oxidative polymerization process to synthesize PPy on the surface of IPU films. After the ultrasonic treatment in ethanol, the conductive IPU films were obtained.

*Characterization*

The nanostructure of the fabricated templates and IPU films was revealed by a field emission scanning electron microscope (FESEM) (SU8010, Hitachi). Reflection spectra of the SC films were measured using a fiber-optic spectrometer (NOVA, Ideaoptics). The stress-strain test was carried out by universal testing machine (6800, Instron). The resistance of the conductive IPU films was measured by a digital multimeter (DMM6500, Keithley).


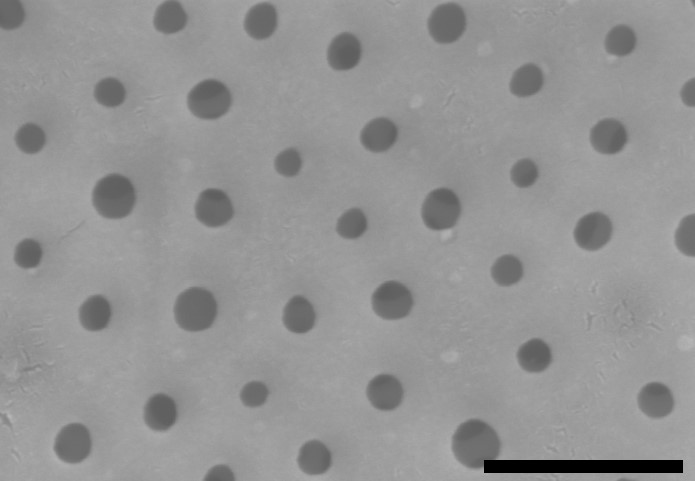


**Figure S1**. SEM image of the PU film fabricated with polydisperse silica colloidal nanoparticles. Scale bar is 500 nm.

**
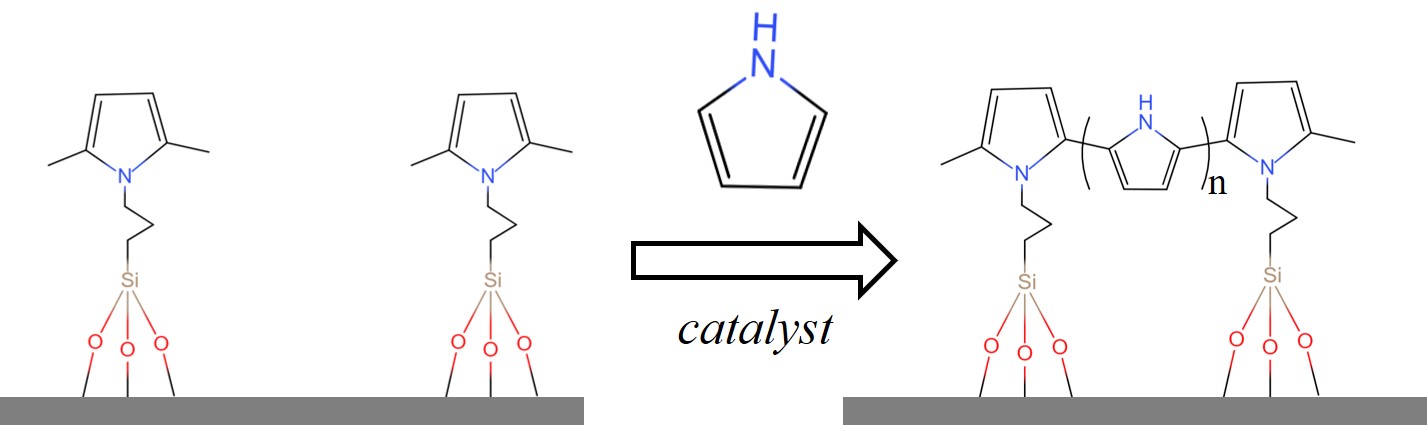
**

**Figure S2**. N-(3-trimethoxysilylpropyl) pyrrole was first grafted onto the surface of the film through covalent bonding. Then under the catalysis of Iron(III) p-toluene sulfonate hexahydratein, pyrrole monomer was polymerization into polypyrrole.


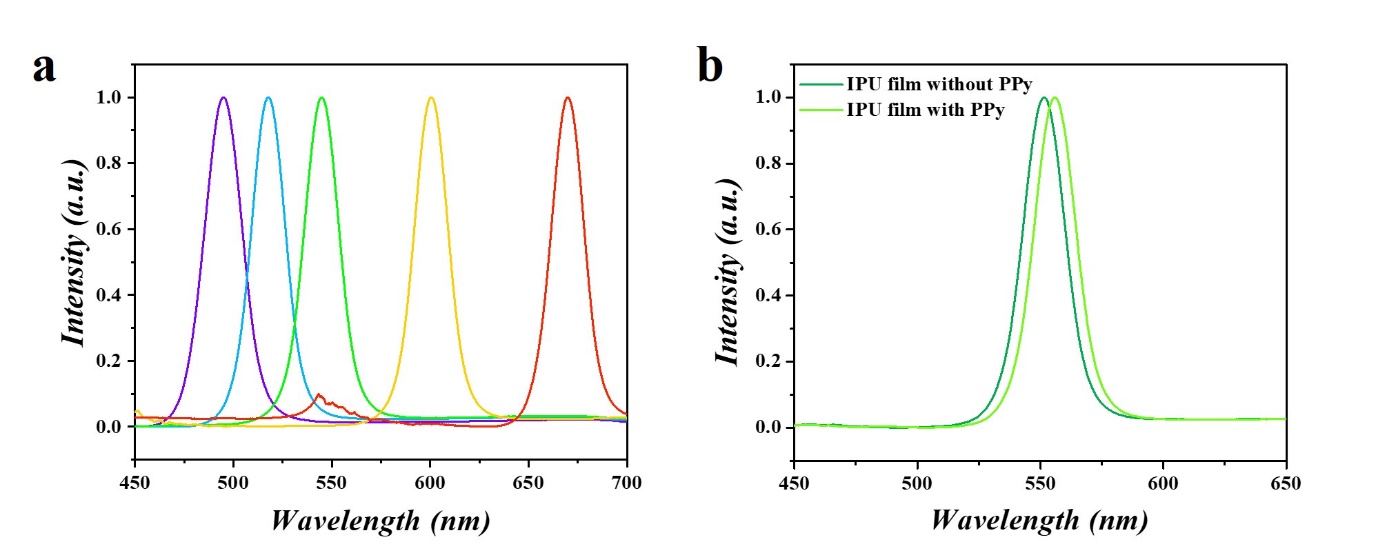


**Figure S3**. Optical properties of the SC films. a) Reflection spectra of the SC film fabrication by silica colloidal nanoparticles with diameters of 215, 225, 237, 260, and 290 nm, respectively. b) Reflection spectra showed the reflection peak shift after the PPy polymerization.


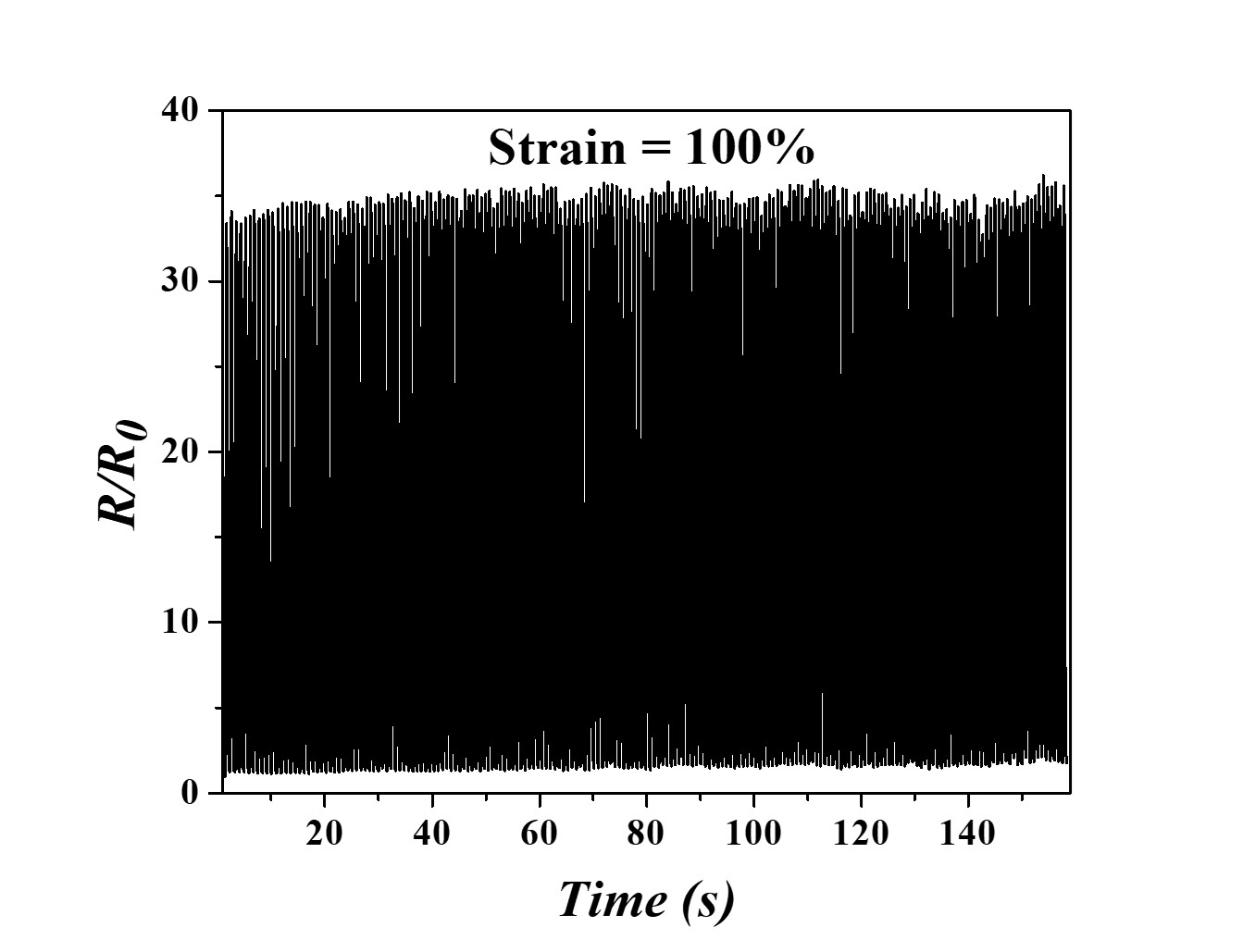


**Figure S4**. Conductive stability and repeatability of the conductive SC film. The relative resistance change of the conductive SC film under 100% tensile strain with 200 repetitive cycles.


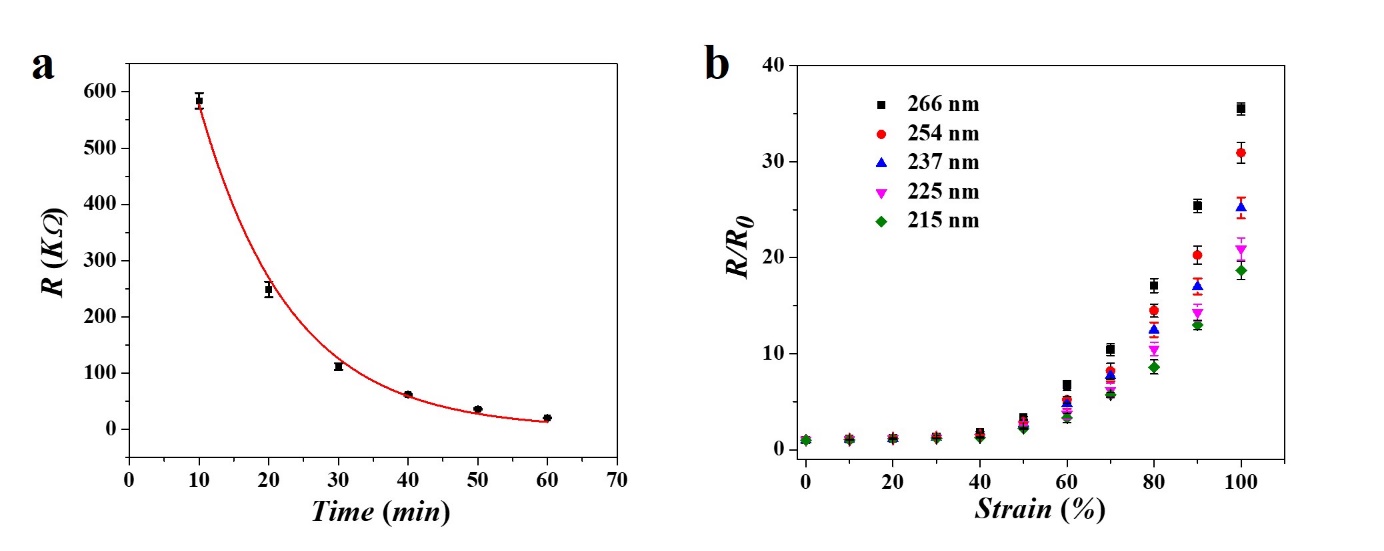


**Figure S5**. Conductive properties of the conductive SC film. a) The resistance of the conductive SC film under different polymerization time of PPy. b) Relative resistance change during stretching for films fabricated with different silica colloidal nanoparticle diameters.


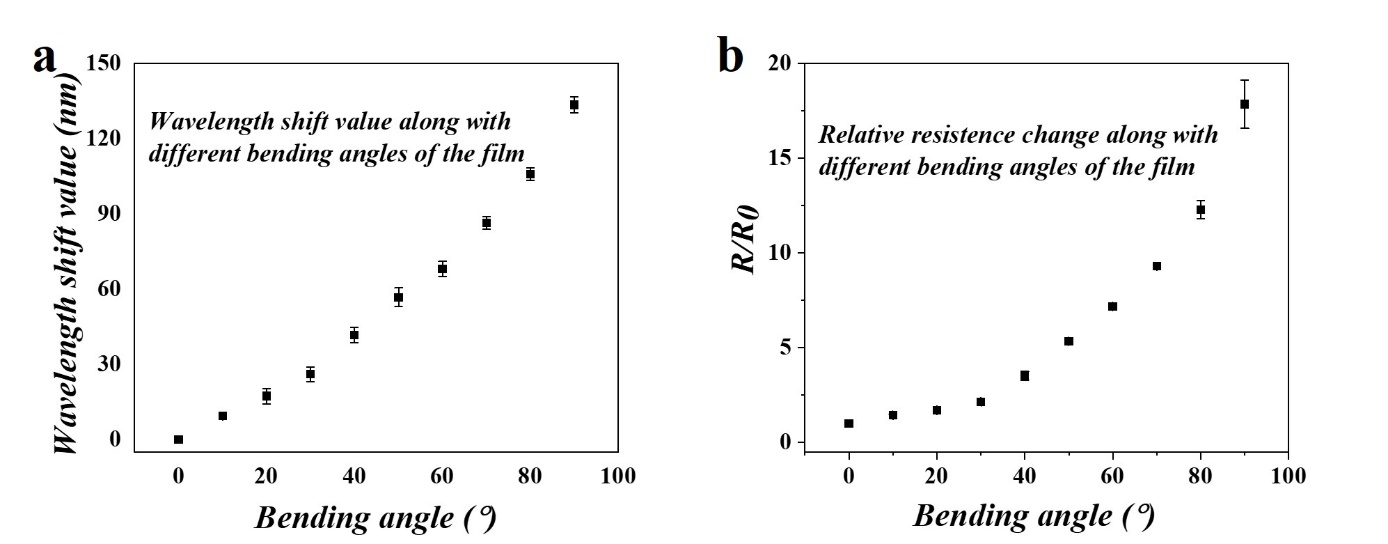


**Figure S6**. Dual-signal response to different bending angles of finger. a) The reflection wavelength shift values of the conductive SC film in response to different bending angles of finger. b) The relative resistance change of the conductive SC film in response to different bending angles of finger.
